# Poorly differentiated thyroid carcinoma: a retrospective clinicopathological study

**DOI:** 10.11604/pamj.2015.21.137.6720

**Published:** 2015-06-22

**Authors:** Ghofrane Salhi Cherkaoui, Amal Guensi, Sara Taleb, Malika Ait Idir, Najwa Touil, Rita Benmoussa, Zaineb Baroudi, Nabil Chikhaoui

**Affiliations:** 1 Nuclear Medicine Department, Ibn Rochd University Hospital, Université Hassan II, Casablanca, Morocco; 2 Emergency Radiology Department, Ibn Rochd University Hospital, Université Hassan II Casablanca, Morocco

**Keywords:** Thyroid neoplasms, poorly differentiated thyroid carcinoma, diagnosis, treatment

## Abstract

Poorly differentiated thyroid carcinoma (PDTC) is an independent thyroid cancer histotype. In spite of its scarcity, it represents the main cause of death from non-anaplastic follicular cell-derived thyroid cancer. However, given the newness of this entity, few data are available on its clinical behaviour and no explicit consensus sets its treatment. To report the experience of a tertiary medical centre in morocco with PDTC over a period of 7 years. Retrospective study selecting all patients treated for thyroid carcinoma in Nuclear Medicine Department of a tertiary medical centre in Casablanca over seven years period. Patient's files were reviewed for background data, clinico-pathological characteristics, treatment and outcome. Seven patients were included in the study. Patient's average age was 60 years old (30-81) including six women and one man. All patients underwent a total thyroidectomy completed by cervical lymph node dissection in 57% of cases. Mean primary tumour size was 4cm (1-9cm). Patients were classified pT3 in 70% of cases, pT1 and pT2 in 15% each. Vascular invasion was found in 85% of cases. Pathological subtypes found were “insular carcinoma” in 85% of cases. Radioiodine therapy (RIT) was indicated in all cases. Follow-up period ranged between 10 months and 6 years. It showed a complete remission in 57% of cases, persistent disease in 28% of cases and a progressive disease in 15% of cases with a local recurrence. To date, the survival rate is 85%. PDTC is an aggressive thyroid cancer histotype. Treatment remains surgical followed by RIT if the tumour is radioavid. Multimodality therapy is indicated depending on the case and close monitoring is always indicated given the high risk of relapse.

## Introduction

Poorly differentiated thyroid carcinoma (PDTC) is a rare and independent thyroid cancer histotype. It was classified as a variant of well differentiated thyroid carcinoma (WDTC) until its recognition in 2004 by the World Health Organisation (WHO) as a distinct pathologic entity. It represents a heterogeneous group which clinical and histological features are not suitable to WDTC or Anaplastic carcinoma (AC). It's defined as “a follicular-cell neoplasm that shows limited evidence of structural follicular cell differentiation and occupy both morphologically and behaviourally an intermediate position between differentiated (follicular and papillary carcinomas) and undifferentiated (anaplastic) carcinoma” [1, 2]. In 2006, the international conference held in Turin - Italy - added more rigorous criteria known as “Turin criteria” to reach diagnosis standardization [2, 3]. PDTC definition includes both differentiation loss and high grade features. It is divided in three pathological subtypes: solid, insular, and trabecular [2–4]. In spite of its scarcity, PDTC represents the main cause of death from “nonanaplastic follicular cell-derived thyroid cancer” [5]. However, given the newness of this entity, few data are available on its features and none of them involves patients in North Africa. To date, there is no explicit consensus to sets PDTC patients’ management. This study intended to report the experience of a tertiary medical centre in Morocco by reporting patients’ features, clinical stage at the time of diagnosis, the treatment applied, occurrence of distant metastases and the survival.

## Methods

The study involved all patients diagnosed histologically with PDTC among 3000 patients treated for thyroid carcinoma in Nuclear Medicine Department Ibn-Rochd University Hospital of Casablanca- Morocco- between January 2007 and December 2013. Histological diagnosis was based on “Turin criteria” [6]. The patients’ medical data was analysed retrospectively. Files were reviewed for background data, clinico-pathological characteristics, treatment and outcome. **Collected data:** demographic data: gender, age; the revealing clinical sign; surgical treatment; histological features: histotype, tumour size, capsular invasion, vascular emboli, loco-regional invasion and lymph node metastases; patients were staged according to the tumour node metastasis (TNM) classification-2010 [7]; Iodine free diet and the thyroid hormone withdrawal were applied for at least four weeks prior to 131 iodine therapy and serum thyroglobulin (Tg) measurements. TSH level was kept above 30µUI/ml; therapy dose was 3700 or 5550 MBq (100 or 150mCi) depending on the Tg levels and the presence of metastases, 131I whole body scan (WBS) was performed two days after radioiodine therapy to evaluate thyroid remnant and distant metastases. Additional imaging modalities were requested depending on WBS results. Follow-up by means of cervical examination, neck ultrasonography. Serum Tg checking and 131l WBS (with 111 MBq-3mCi-of 131iodine) were done after thyroid hormone withdrawal 6 to 9 months after RI. Interval between two therapies was higher than 6 months in repeated therapies.

According to the results, patients were ranged in three groups: complete Remission: patients who completely responded to therapy with normal WBS and undetectable serum Tg (stimulated Tg levels below 1ng/ml); residual disease: positive or negative WBS results with positive Tg level reduced below the initial measurement; progressive disease: patients who are considered as non responders to therapy with positive or negative WBS results with unchanged or increased Tg levels. Cumulative dose was reported in each case.

## Results

Among the 3000 thyroid carcinoma patients admitted in our department during the study period, seven patients were diagnosed with PDTC, 0.23% of our material. Patient's average age was 60 years old (30- 81) including six women and one man. Four patients (57%) lived in non coastal areas. No radiation exposure history was reported. A cervical mass was noted in all cases at the admission without dysthyroidism signs. All patients were qualified for operative treatment. They underwent a total thyroidectomy, completed by cervical lymph node dissection in four cases (57%) showing regional lymph node involvement in all of them. The mean primary tumour size was 4 cm (1-9cm). According to AJCC staging, patients were classified pT3 in 70% of cases (capsular and peri-thyroidal fat invasion), and pT1 and pT2 in 15% each. Vascular invasion was found in 6 cases (85% of cases). Pathological subtypes found were “insular carcinoma” in 6 cases and “Trabecular carcinoma” in one case. The endogenous TSH stimulated Tg level prior RIT ranged between 3 and 65ng/ml in five cases (70%) and above 100ng/ml in three cases (30%) among them two had distant metastasis. Anti-thyroglobulin antibodies were not dosed. WBS results after the first RIT showed a 131I neck uptake pointing the existence of thyroid remnants in all cases. It was associated with thoracic uptakes in one case, and both lung and bone (skull) uptakes in one another. Cumulative dose of radioiodine therapy was equal to 3700 MBq (100mCi) in four cases and 9250 MBq (250mCi) in three cases. The indication of successive doses of RIT resulted from the presence of radioavid metastasis. Follow-up period ranged between 10 months and 6 years. It showed: complete remission in four cases, half of them had a tumour staged T1-2 and all of them had a Tg level 100ng/ml). In one case, the WBS and CT revealed local and cervical node recurrences. A third RIT is scheduled after the surgery. In the other, the WBS still show thoracic uptake after two RIT courses. CT scan showed bilateral pulmonary nodules. Scheduled for a third RIT; Progressive disease in one case with a local recurrence. This patient was a woman of 55 years old with a tumour staged initially: pT3 N1b M+ (bone and lungs) with initial Tg measurement equal to 42 ng/ml. She died one week after the second RIT. Last Tg level was 4866ng/ml. The latest consultation showed a survival rate of 85%. No statistical analysis has been performed due to the low number of cases.

## Discussion

This study group intended to report the experience of a tertiary Moroccan medical centre with PDTC, trying to add more information on its features. According to previous series, its incidence range between 4-7% among patients with thyroid carcinomas [5, 8]. The frequency of this tumour reaching 15% of thyroid carcinomas in some series in northern parts of Italy suggests the presence of genetic and /or environmental risk factors such as iodine deficiency in addition to radiation exposure [8]. In our study the incidence was 0.23% and iodine deficiency may be considered as a risk factor, since more than half patients lived in non coastal regions, which are known for their iodine deficiency. PDTC should be considered a negative prognostic factor in patients with thyroid carcinoma.

Histologically, its diagnosis is based on differentiation loss (non follicular - non papillary growth pattern), and high grade features (invasive growth - high mitotic index and necrosis). PDTC displays predominant solid, insular, or trabecular growth patterns which can coexist with follicular, papillary or anaplastic carcinoma component [4, 9]. Indeed, according to the current models of tumour progression (WDTC evolving into PDTC then anaplastic carcinoma), the co-existence in the same tumour of WDTC with PDTC component may be seen. However, for PDTC diagnosis assessment, the majority of cell tumours must exhibit poorly differentiated features. Even though consensus conferences did not state a precise percentage yet, the quote adopted by authors is 75% [3, 10]. It is however important to report the presence of PDTC component in an otherwise conventional WDTC since the high grade component may drive the prognosis [3]. In the current study, the mean age was 60 years with a high ratio of women (87%). These data support the fact that PDTC often occurs in the female gender in older age, most often in the sixth decade, consistently with the literature [5, 8, 11]. This study showed also the local and the distant aggressiveness of PDTC. When diagnosed, it was already at an advanced stage of disease, with extrathyroidal extension in 70% of cases, regional lymph nodes metastasis in more than half of cases and distant metastases in 43% of cases.

According to previous studies and mainly the large series of Ibrahimpasic et al. [5], PDTC presents more frequently with invasive disease associated to lymph node metastasis. Distant spread is more commonly present in PDTC than WDTC with the same areas of predilection, namely bone and lungs. However, distant metastases may interest exceptionally unusual sites such as the skin, liver, ovaries, retro-peritoneum and intraocular [12, 13]. To date, there are no standard guidelines for PDTC management due to the previous lack of standard diagnostic criteria. Primary treatment commonly implemented remains the same than WDTC. It associates total thyroidectomy and lymph node dissection whenever feasible, since regional node metastasis are relatively frequent [14]. Adjuvant therapy such as RIT, External beam radiotherapy (EBRT) and chemotherapy remain poorly established. In the current study, all patients underwent total thyroidectomy and RIT. Neck dissection depended on preoperative and intra-operative determinations. RIT is a relatively harmless therapy which showed its effectiveness in WDTC. In PDTC, its indication is controversial. Even though poorly differentiated cells have the ability to trap radioiodine in more than 80% of cases [9, 14], including distant metastasis at the time of diagnosis [5, 15], 15% of cases has a decreased iodine uptake, and consequently a limited RIT value. In our series, all cases showed radioavidity. Molecular pathways suggest that this iodine dysregulation of function may be due to BRAF mutation [16]. In addition, no statistical analysis did demonstrate RIT benefit on patient outcomes neither on their survival [5]. External beam radiotherapy (EBRT) utility in PDTC is still questionable. It is however indicated depending on locoregional recurrence risk, especially patients with positive margins, gross residual disease and regional lymph node metastasis with no distant metastasis [5]. It can be also used as a palliative treatment in bony metastases in whom RIT has failed [17]. Furthermore, Chemotherapy is reserved for inoperable cases. It contributes theoretically to loco regional control by improving respectability or by reducing disease progression [14]. In PDTC, patients’ overall five years survival range between 50 - 89% [8, 15, 18, 19].

In the study of Katsuhiro et al. which included 29 patients with PDTC, the specific 10 years survival was 89.9%. Volante et al. introduced a scoring system which ranks patients in three categories depending on three prognosis factors: patients age (> 45 years), mitotic index (>3/10HPF) and the presence of necrosis [18, 20] However, this scoring system is biased; it includes histological factors that are also considered as diagnostic marks. In the current study, unfavourable outcome seemed to be related to lymph node, distant metastases and to local recurrence. Univariate analysis in consecutive studies have included age, the high TNM stage, extrathyroidal extension, positive margins and distant metastasis as factors associated with unfavourable outcome [4, 21].

## Conclusion

PDTC is an aggressive thyroid cancer histotype with high tendency to extrathyroidal extension, regional node and distant metastases. Treatment remains the same than WDTC with a neck dissection whenever feasible followed by RIT if the tumour is radioavid. A close monitoring is required to improve its outcome. **Strengths and limitations of this study:** to our knowledge, this is the first study treating the subject of Poorly Differentiated Thyroid Carcinoma in Morocco and in North Africa. This study is retrospective, monocentric based only on medical records. It is difficult to draw substantial conclusions from a small study group, with a short follow-up period.
